# Much higher prevalence of keratoconus than announced results of the Gutenberg Health Study (GHS)

**DOI:** 10.1007/s00417-023-06132-y

**Published:** 2023-06-14

**Authors:** Susanne Marx-Gross, Achim Fieß, Thomas Münzel, Philipp Sebastian Wild, Manfred Elmar Beutel, Irene Schmidtmann, Karl Johannes Lackner, Norbert Pfeiffer, Alexander Karl-Georg Schuster

**Affiliations:** 1 Department of Ophthalmology, University Medical Center of the Johannes Gutenberg-University Mainz, Langenbeckstrasse 1, 55131 Mainz, Germany; 2 Artemis Augenzentrum Wiesbaden, Wiesbaden, Germany; 3 MVZ of University Medical Center of the Johannes Gutenberg-University Mainz GmbH, Mainz, Germany; 4 Center for Cardiology , Cardiology I, University Medical Center of the Johannes Gutenberg-University Mainz, Mainz, Germany; 5 Preventive Cardiology and Preventive Medicine – Department of Cardiology, University Medical Center Mainz, Mainz, Germany; 6 Center for Thrombosis and Hemostasis, University Medical Center Mainz, Mainz, Germany; 7 German Center for Cardiovascular Research (DZHK), Partner Site Rhine-Main, Mainz, Germany; 8 Institute of Molecular Biology (IMB), Mainz, Germany; 9 Department of Psychosomatic Medicine and Psychotherapy, University Medical Center of the Johannes Gutenberg University Mainz, Mainz, Germany; 10 Institute for Medical Biometry, Epidemiology and Informatics (IMBEI), Mainz, Germany; 11 Institute of Clinical Chemistry and Laboratory Medicine, University Medical Center of the Johannes Gutenberg-University Mainz, Mainz, Germany

**Keywords:** Keratoconus, Pentacam, Epidemiology, Prevalence, Associated factors

## Abstract

**Abstract:**

Keratoconus appears to be a rare corneal disease with a prevalence previously estimated at 1:2000. The aim of our study was to investigate the prevalence of keratoconus in a large German cohort and to evaluate possible associated factors.

**Method:**

In the population-based, prospective, monocentric cohort study, Gutenberg Health Study, 12,423 subjects aged 40–80 years were examined at the 5-year follow-up. Subjects underwent a detailed medical history and a general and ophthalmologic examination including Scheimpflug imaging. Keratoconus diagnosis was performed in two steps: all subjects with conspicuous TKC analysis of corneal tomography were included in further grading.

Prevalence and 95% confidence intervals were calculated. Logistic regression analysis was carried out to investigate association with age, sex, BMI, thyroid hormone, smoking, diabetes, arterial hypertension, atopy, allergy, steroid use, sleep apnea, asthma, and depression.

**Results:**

Of 10,419 subjects, 75 eyes of 51 subjects were classified as having keratoconus. The prevalence for keratoconus in the German cohort was 0.49% (1:204; 95% CI: 0.36–0.64%) and was approximately equally distributed across the age decades. No gender predisposition could be demonstrated.

Logistic regression showed no association between keratoconus and age, sex, BMI, thyroid hormone, smoking, diabetes, arterial hypertension, atopy, allergy, steroid use, sleep apnea, asthma, and depression in our sample.

**Conclusion:**

The prevalence of keratoconus disease in a mainly Caucasian population is approximately tenfold higher than previously reported in the literature using latest technologies (Scheimpflug imaging). Contrary to previous assumptions, we did not find associations with sex, existing atopy, thyroid dysfunction, diabetes, smoking, and depression.

**Supplementary Information:**

The online version contains supplementary material available at 10.1007/s00417-023-06132-y.


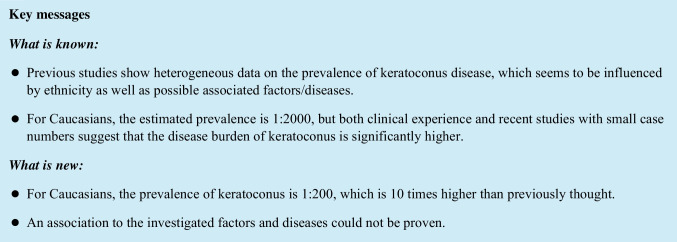


## Introduction

Keratoconus is a corneal disease, which progresses within adolescence and early adulthood. The corneal irregularity and progressive thinning can cause refractive error, which cannot be corrected easily by glasses and leads to reduced vision. Keratoconus patients may use contact lenses, but in very advanced cases, only a corneal transplant can improve vision. Nowadays, there are treatment options, for example, corneal cross-linking, to stop the progression of keratoconus in an early stage [1].

Nevertheless, vision is often reduced and quality of life may be impaired [2]. The deterioration of vision often requires adaptation to daily life and professional development [3]. Therefore, it is essential to know the disease burden in order to assess the need for medical care.

Keratoconus appears to be a rare corneal disease with a prevalence previously estimated at 1:2000 [4]. However, in recent relatively small studies, a higher prevalence was found. Prevalences are reported from 1: 6000 for US citizens, 1:1000 for Israeli adolescents up to 1:20 for Saudi-Arabian Children [5–8]. Newer studies detected prevalence estimates of 1:191 in New Zealand high school students [9]. A prevalence up to 1:84 was detected for young Australian adults [10]. The prevalence seems to be dependent on ethnicity. Recent analysis of approximately 142,000 patient records in Ghana showed a lower prevalence of 1:2000 [11]. A gender predisposition has been discussed: Keratoconus seems to occur more often in male subjects, but some newer results found no sex predisposition [8, 12]. It is difficult to compare different studies, as they differ not only in sample size but also in diagnostic methods, disease definition, and sample selection [8].

Technologies such as Scheimpflug imaging allow the detection of irregularities of the front and back of the cornea as well as pachymetric parameters of the cornea.

Up to now, the hypotheses for the origin and progress of keratoconus are contradictory. Keratoconus has been associated with other disorders such as atopia, thyroidal disorders, which could be hypothyreosis and hyperthreosis, and hypercortisolism [13–15], while diabetes and smoking are described in the literature as potentially protective factors [16–18].

Thus, the aim of our study was to investigate the prevalence of keratoconus in a large German cohort using Scheimpflug technology, and to detect possible associated factors.

## Materials and methods

The Gutenberg Health Study (GHS) is a prospective population-based cohort study that includes women and men aged 35 to 74 years at baseline who reside in the city of Mainz (194,425 inhabitants) or in the rural district of Mainz-Bingen (210,867 inhabitants). Out of these, a random sample of 35,000 subjects was stratified 1:1 for sex, 1:1 for place of residence (Mainz and Mainz-Bingen, rural vs. urban and equally for the 4 age decades via the local residents’ registration offices). The study cohort of 15,000 was drawn in waves of equal stratification to allow subsample analyses. The baseline examination was conducted between 2007 and 2012 in the total cohort of 15,010 participants. The 5-year follow-up investigation of the cohort was carried out between 2012 and 2017 and 12,423 subjects aged 40–80 years were examined again.

Exclusion criteria were mentally or physically ill persons who were unable to visit the study center or participate in study-related examinations, and persons with insufficient knowledge of German [19].

All subjects underwent a detailed medical history and an internal and ophthalmological examination including Scheimpflug imaging (Pentacam®, Oculus, Wetzlar, Germany) at the 5-year follow-up examination. Keratoconus diagnosis was performed in two steps: all subjects with conspicuous TKC analysis of corneal tomography were included in the further diagnosis (*n* = 662). An additional group without conspicuous TKC findings were included in further diagnosis as well (*n* = 50). In all these subjects, a masked double grading for keratoconus was performed on the corresponding Scheimpflug imaging (Pentacam®, Oculus, Wetzlar, Germany) by two experienced ophthalmologists. They analyzed the axial/sagittal images of the anterior and posterior corneal surface, pachymetry, and keratoconus indices as well as the Belin/Ambrósio Enhanced Ectasia Display. In case of different findings, a third ophthalmologist was consulted. First, we used the automatic analysis of Pentacam®, which is based on the Amsler classification. Only images with high quality were used.

Prevalence and 95% confidence intervals for keratoconus were calculated for the whole sample, as well as stratified by sex and age decade. Quantitative ocular characteristics were described by median, minimum, maximum, and quartiles, stratified by presence of keratoconus and side. Box plots were used for visualization.

Univariable logistic regression analyses were conducted to investigate possible associations with age, sex, body-mass-index (BMI), thyroid-stimulating hormone (TSH), smoking, diabetes, arterial hypertension, atopy, allergy, steroid use, sleep apnea, and asthma. A possible association with depression were tested in a separate exact logistic regression model with depression as the dependent variable and keratoconus as the independent variable. Depression was defined as a PHQ-9 value ≥ 10 [20]. Odds ratios with 95% confidence intervals and *p*-values are presented. All statistical analysis were computed using SAS 9.4.

## Results

Of 10,419 subjects, 75 eyes of 51 subjects were classified as having keratoconus. The prevalence for keratoconus in this German cohort was 0.49% (1:204; 95% CI: 0.36–0.64%) and was approximately equally distributed across the 4 age groups (40–49 years: 0.49%, 50–59 years: 0.55%, 60–69 years: 0.51%, 70–80 years: 0.44%). No gender difference could be demonstrated (male/female: prevalence of 0.52%/0.45%) (Table 1).

**Table 1:** Prevalence of keratoconus disease in the German Gutenberg Health Study (2012*–*2017). Topographical keratoconus classification and grading was used to determine keratoconus cases

Sex	Age [decade]	*n*	Keratoconus	Prevalence [%]	Exact 95% CI lower limit [%]	Exact 95% CI upper limit [%]
All	40–80	10,419	51	0.49	0.36	0.64
All	40–49	2368	13	0.55	0.29	0.49
	50–59	2959	15	0.51	0.28	0.83
	60–69	2826	13	0.46	0.25	0.79
	70–80	2266	10	0.44	0.21	0.81
Male	40–80	5352	28	0.52	0.35	0.76
Female	40–80	5067	23	0.45	0.29	0.68

**Fig. 1:**
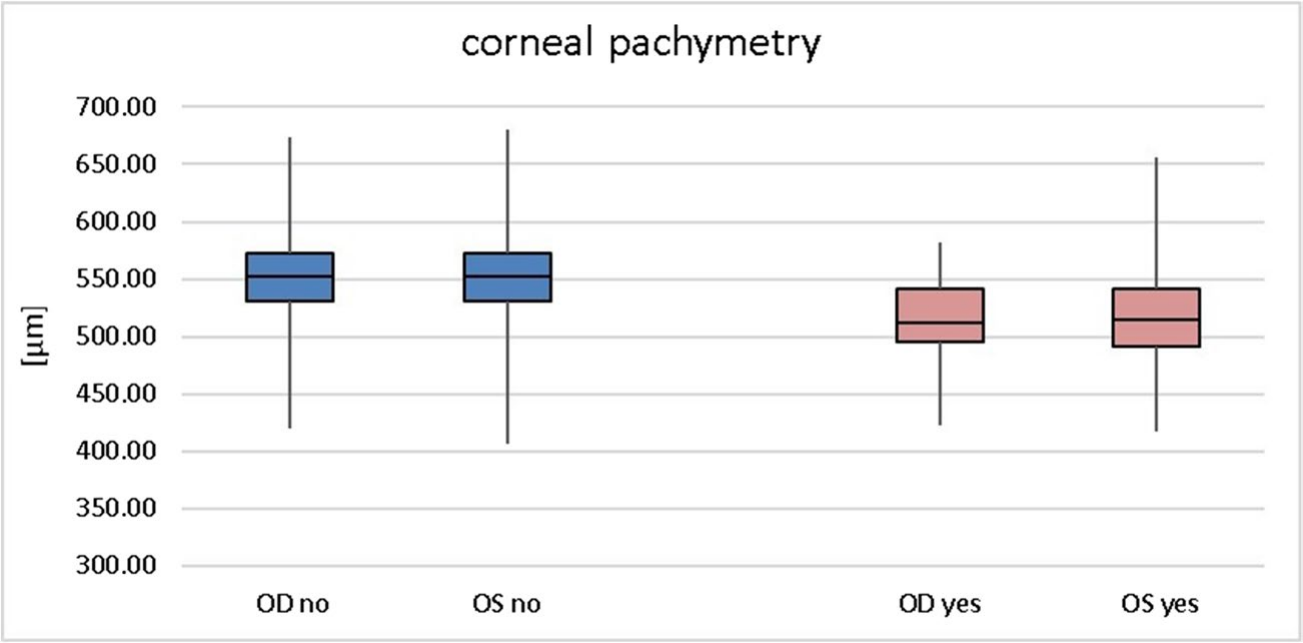
Central corneal thickness of subjects with (yes) and without (no) keratoconus in right (OD) and left (OS) eyes. Data from the Gutenberg Health Study (2012–2017)

**Fig. 2:**
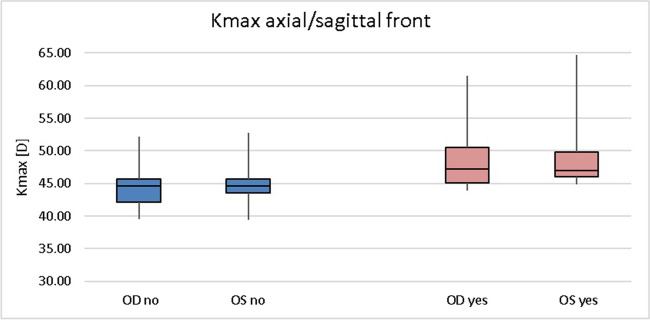
Maximal corneal power (Kmax) of subjects with (yes) and without (no) keratoconus regarding Kmax (D) of right (OD) and left (OS) eyes. Data from the Gutenberg Health Study (2012–2017)

With respect to ocular parameters, a more myopic sphere and a higher cylindric power was present in those subjects with keratoconus (Table 2). Considering the median central corneal thickness, subjects with keratoconus had a thinner cornea than those without keratoconus, although the minimum did not show large differences, as in the non-keratoconus group, there had been some subjects after laser refractive surgery as well (Fig. 1), although the K max is higher in the keratoconus group (Fig. 2) Table 3.

**Table 2:** Ocular characteristics of subjects with (yes) and without (no) keratoconus regrading best corrected visual acuity (BCVA, log MAR), sphere and cylinder (D) of right (OD) and left (OS) eyes. Data from the Gutenberg Health Study (2012–2017)

		Median	Q3	Max	Min	Q1
Visual acuity	No OD	0.10	0.20	3.00	− 0.30	0.00
	No OS	0.10	0.20	3.00	− 0.30	0.00
	Yes OD	0.20	0.30	0.60	− 0.10	0.10
	Yes OS	0.20	0.30	0.70	0.00	0.10
Sphere (D)	No OD	0.00	1.00	11.25	− 25.00	− 1.00
	No OS	0.00	1.25	11.25	− 20.75	− 1.00
	Yes OD	0.50	1.25	3.00	− 11.25	− 0.50
	Yes OS	0.00	1.38	4.00	− 11.25	− 1.50
Cylinder (D)	No OD	− 0.50	− 0.25	0.00	− 9.75	− 0.75
	No OS	− 0.50	− 0.25	0.00	− 9.75	− 0.75
	Yes OD	− 0.50	− 0.25	0.00	− 3.25	− 1.00
	Yes OS	− 0.63	− 0.50	0.00	− 8.50	− 1.63

**Table 3:** Association analysis of keratoconus with systemic and anthropometric parameters. Data from the Gutenberg Health Study (2012–2017, n = 10.419). Univariable logistic regression analysis was computed

Associated factors	*OR*	95% CI: lower	95% CI: upper	*p*-value
Sex female vs. male	0.87	0.50	1.52	0.63
Age (per year)	0.99	0.97	1.02	0.56
Socioeconomic status: high (> 14) vs. medium	0.95	0.52	1.75	0.28
Socioeconomic status: low (> 7.8) vs. medium	1.78	0.80	3.96	0.12
BMI (< 18.5 vs. [25.0;30.0])	3.14	0.42	23.7	0.23
BMI (18.5;25.0 vs. [25.0;30.0])	0.92	0.48	1.75	0.32
BMI (≥ 30 vs. [25.0;30.0])	0.89	0.44	1.80	0.30
TSH (per unit)	0.88	0.57	1.35	0.56
Smoking	0.72	0.31	1.69	0.45
Diabetes	1.02	0.40	2.58	0.97
Hypertension	1.19	0.68	2.08	0.54
Atopy	2.02	0.62	6.54	0.24
Allergy	1.00	0.57	1.74	0.99
Steroids	0.46	0.06	3.36	0.45
Sleep apnoea	0.62	0.19	2.01	0.18
Asthma	1.23	0.38	3.98	0.73
Arthropathy	1.40	0.63	3.14	0.87
Depression PHQ ≥ 10	2.21	0.89	4.20	0.86

Logistic regression showed no association between keratoconus and age, sex, BMI, thyroid-stimulating hormone (TSH), smoking, diabetes, arterial hypertension, atopy, allergy, steroid use, sleep apnea, asthma, and depression in our sample (Table 1).

## Discussion

The prevalence of keratoconus varies in the literature between 1:20 and 1:2000 in different regions and study designs [4–9]. Thus, to assess the burden of disease, it is necessary to estimate the prevalence of the disease in large population-based studies. In our experience, the numbers of patients in ophthalmologic practices and clinics are increasing significantly, suggesting that the prevalence may be much higher.

Recently, new therapeutical options such as corneal cross-linking may allow to decelerate or even to stop the progression of keratoconus and to lower the risk of visual impairment [21]. Diagnostic approaches include corneal topography and corneal tomography, which detect the disease in early subclinical stages [22]. However, only a limited number of studies have used these technologies to estimate prevalence [5, 23] while previous studies often used questionnaires, refractive measurements, or only topography of the corneal front [24–27]. These studies often examined only small cohorts up to 987 subjects [4, 5, 23, 24, 27]. Several studies with large study populations use health claim data in retrospective database analyses [6, 7, 12, 28]. Limitations in these studies are risks of miss-classification due to non-standardized examination techniques and unclear stage of disease. Thus, form fruste keratoconus and earlier stages could be missed resulting in an underestimation of prevalence.

To determine prevalence, some research groups exclusively investigated young populations (6–40 years of age) [5, 6, 23, 24, 27, 28]. This may lead to an underestimation of the prevalence, as keratoconus development may not be completed yet [29]. Studies in this age group based on database analysis alone reported prevalence estimates of 0.03 to 0.26% [6, 11, 12, 28]. One study reported a markedly higher prevalence of 4.79% in young people using Scheimpflug imaging, but this study recruited 522 study participants in multiple non-ophthalmic emergency departments in Saudi Arabia, thus, does not reflect a population-based approach [5]. The research group led by Hashemi et al. also identified a high prevalence of up to 4% [30] in an Arab population, same in a very young cohort in Australia and New Zealand. They analyzed young adults in a small cohort and estimated a high prevalence of 1:84 and 1:191 in a mixed cohort [9, 10].

Similarly, the prevalence also appears to be higher in the Indian population, been estimated at 2.3% in those over 30 years of age [25], while in the Asian population, the prevalence was estimated at 0.03% based on a retrospective database approach [12] and 0.9% in the Beijing Eye Study [26]. Within the USA, Woodword showed a higher prevalence among Asians and Arabs compared to Caucasians [17]. Comparable Pearson et al. showed in a retrospective hospital-based study in the UK that the prevalence in the age group below 40 years was four times higher in Asians compared to Caucasians [31].

The population-based prevalence of Keratoconus was estimated in a Norwegian register study using data from the Norwegian Patient Registry and resulting in a prevalence estimate of 0.19% [32].

In our predominantly Caucasian cohort, the prevalence was 0.49%, which was much lower than in the Arab population and approximately 2–3 times higher than in the analyses using health claim data. Newer studies for UK detected a prevalence of 0.15% and in a subanalysis of citizens of Colorado showed a prevalence of approximately 0.51% which is similar to our study [33].

Nevertheless, we have considered potential limiting factors in our study approach.

We conducted a population-based study and systematically examined a large Caucasian cohort at age 40 years and above regarding keratoconus prevalence using modern Scheimpflug imaging and consecutive double grading of conspicuous cases. We chose a cohort size of about 15,000 participants, who were not only examined ophthalmologically but also with regard to internal and mental disorders in order to be able to assess disease progression in 5-year follow-ups. In general, keratoconus is a rarer disease, so that despite the large cohort, the number of cases with 75 eyes is not too large. But the study design, prospective, randomized, stratified, and the differentiated analysis of the study results using Scheimpflug imaging enables a reliable statement on the prevalence and association with possible risk factors. The recruitment efficacy proportion at baseline was 55.5%. A total of 82.6% of the study participant did undergo the 5-year follow-up examination as well. At the time of study examination, all subjects were over 40 years of age. Thus, it can be assumed that all subjects with keratoconus had already developed the disease.

The extent to which keratoconus is associated with other factors as age, sex, BMI, thyroid hormone, smoking, diabetes, arterial hypertension, atopy, allergy, steroid use, sleep apnea, asthma, and depression has been investigated by various working groups. The results were very heterogeneous. For our cohort, we could not detect any accumulation. Only in the context of depression have we detected a tendency to cluster in the keratoconus group, but without clinical significance.

A detailed placement of our nonsignificant associated factor results in the current literature context is provided in the supplement.

## Conclusion

In conclusion, the prevalence of keratoconus in the German population is approximately tenfold higher (0.49%) than previously reported in the literature using latest technologies, namely Scheimpflug imaging. Contrary to previous assumptions, keratoconus is more likely in males; this was not shown in our data. In addition, we did not find associations with existing atopy, thyroid dysfunction, diabetes smoking, or depression.

## Supplementary Information

Below is the link to the electronic supplementary material.

Supplementary file1 (DOCX 61.7 KB)

## Declarations

The study was presented as FR10-01 on 30.09.2022 at 120. Congress DOG, Berlin, Germany.
