# Supplementary material for: Much higher prevalence of keratoconus than announced results of the Gutenberg Health Study (GHS)
Source: Graefes Arch Clin Exp Ophthalmol. 2023 Jun 14;261(11):3241–7. doi: 10.1007/s00417-023-06132-y (PMC10264871; doi:10.1007/s00417-023-06132-y)
Supplement: Supplementary file 1 — Supplementary file1 (DOCX 61.7 KB) [file 417_2023_6132_MOESM1_ESM.docx]

**Supplement to the discussion, detailed consideration of the different non-significant parameters:**

Age

In our population of 40- to 79-year-old subjects, there were no differences in prevalence between the different age decades. This is not surprising since keratoconus has disease onset during youth and early adulthood; new incident cases rarely occur after the age of 40 years [1, 2].

Gender

In everyday practice, men appear to be affected more frequently than women are. This was also found by the working group led by Millodot et al., which examined Israeli college students in Jerusalem as well as in the Norwegian register study, in which they found 73% males.[3, 4] Studies on Indian cohorts also detected more male patients [5, 6]. In contrast, a few studies reported that women were more likely to have keratoconus [7, 8]. However, this could not be confirmed in our study. In our cohort there was no gender difference detected (Odds Ratio: 0.93; 95% CI [0.50; 1.52], p-value 0,63), similar to result in other studies [9, 10].

Socioeconomic status

No significant disadvantage for keratoconus patients was detected in our cohort. This is likely due to good medical care with health insurance covering costs of contact lenses and treatment [10, 11].

Depression

Systematic analyses of the association of depression and keratoconus are scarce. In our large population, we did not find any association between keratoconus and depression as well as the large Beijing Eye Study for the Asian population and Woodward et al. [10-12]. We assumed that depression might be more common in keratoconus patients. A study on Greeks have shown that severe keratoconus disease is associated with depression [13].

The question is, whether keratoconus is associated with depression or if keratoconus patients develop depression secondarily. In the Greek population, depression was more prevalent in those with particularly poor visual acuity and pronounced stages of disease [13]. In our cohort best-corrected visual acuity in the keratoconus group was only slightly worse than in the comparison group, so we didn’t find any association with depression. This rather suggests no direct correlation of the two diseases.

BMI and sleep apnea

Some studies found an association with high BMI/obesity and Keratoconus [14] or a combined association of high BMI and sleep apnea [15]. This, we could not confirm in our study. There was no correlation between BMI and Keratoconus disease.

Thyroidal dysfunction

Laboratory chemistry studies and case reports suggested that keratoconus is associated with hypothyroidism [16]. Furthermore, there are case reports in which keratoconus increased significantly during pregnancy in connection with thyroid dysfunction or after thyroidectomy [16, 17]. Both our and other studies could not confirm an association with thyroid dysfunction [18].

Smoking and diabetes

There are some indications in literature that both smokers and subjects with diabetes develop keratoconus less frequently. However, this would require that the patients started smoking at a young age or suffered from diabetes at an early age. One study assumes that the toxic by-products of cigarette smoke cause cross-linking of collagen fibers [19]. However, there is another study showing a positive correlation between the severity of keratoconus and smoking [20].This is in association with our results, no association was found between keratoconus and smoking [8].

With regard to diabetes, it is assumed that there is a stabilizing effect through the glycosylation of the cornea and, thus, a negative correlation with keratoconus is reported [11, 21], which we could not confirm. Similarly, a recent meta-analysis could not prove this either [22]. Kuo et al. found, however, that diabetes obviously causes a stabilization and thus has a positive influence on the severity of the disease [23].

Atopy, allergy and asthma

Some studies have shown associations of atopy, allergy and asthma with keratoconus. In particular, eye rubbing leads to keratoconus progression [24]. It is postulated that increased expression of proteolytic and lysosomal proteins and decreased concentration of protease inhibitors lead to thinning of the cornea [25, 26].

Although the experience from the keratoconus centers as well as the pathogenesis suggests a correlation, we could not confirm an association between atopy or asthma in our study cohort, further we did not have any data about eye rubbing.

**Reference list:**

1. Godefrooij DA, de Wit GA, Uiterwaal CS, Imhof SM, Wisse RP (2017) Age-specific Incidence and Prevalence of Keratoconus: A Nationwide Registration Study. American journal of ophthalmology 175: 169-172 DOI 10.1016/j.ajo.2016.12.015

2. Hashemi H, Asgari S, Mehravaran S, Emamian MH, Fotouhi A (2020) Keratoconus after 40 years of age: a longitudinal comparative population-based study. Int Ophthalmol 40: 583-589 DOI 10.1007/s10792-019-01216-3

3. Millodot M, Shneor E, Albou S, Atlani E, Gordon-Shaag A (2011) Prevalence and associated factors of keratoconus in Jerusalem: a cross-sectional study. Ophthalmic epidemiology 18: 91-97 DOI 10.3109/09286586.2011.560747

4. Kristianslund O, Hagem AM, Thorsrud A, Drolsum L (2021) Prevalence and incidence of keratoconus in Norway: a nationwide register study. Acta ophthalmologica 99: e694-e699 DOI 10.1111/aos.14668

5. Hashemi H, Heydarian S, Yekta A, Ostadimoghaddam H, Aghamirsalim M, Derakhshan A, Khabazkhoob M (2018) High prevalence and familial aggregation of keratoconus in an Iranian rural population: a population-based study. Ophthalmic Physiol Opt 38: 447-455 DOI 10.1111/opo.12448

6. Pearson AR, Soneji B, Sarvananthan N, Sandford-Smith JH (2000) Does ethnic origin influence the incidence or severity of keratoconus? Eye (London, England) 14 ( Pt 4): 625-628 DOI 10.1038/eye.2000.154

7. Hashemi H, Beiranvand A, Khabazkhoob M, Asgari S, Emamian MH, Shariati M, Fotouhi A (2013) Prevalence of keratoconus in a population-based study in Shahroud. Cornea 32: 1441-1445 DOI 10.1097/ICO.0b013e3182a0d014

8. Jonas JB, Nangia V, Matin A, Kulkarni M, Bhojwani K (2009) Prevalence and associations of keratoconus in rural maharashtra in central India: the central India eye and medical study. American journal of ophthalmology 148: 760-765 DOI 10.1016/j.ajo.2009.06.024

9. Hwang S, Lim DH, Chung TY (2018) Prevalence and Incidence of Keratoconus in South Korea: A Nationwide Population-based Study. American journal of ophthalmology 192: 56-64 DOI 10.1016/j.ajo.2018.04.027

10. Xu L, Wang YX, Guo Y, You QS, Jonas JB (2012) Prevalence and associations of steep cornea/keratoconus in Greater Beijing. The Beijing Eye Study. PloS one 7: e39313 DOI 10.1371/journal.pone.0039313

11. Woodward MA, Blachley TS, Stein JD (2016) The Association Between Sociodemographic Factors, Common Systemic Diseases, and Keratoconus: An Analysis of a Nationwide Heath Care Claims Database. Ophthalmology 123: 457-465.e452 DOI 10.1016/j.ophtha.2015.10.035

12. Jonas JB, Wei WB, Xu L, Rietschel M, Streit F, Wang YX (2018) Self-rated depression and eye diseases: The Beijing Eye Study. PloS one 13: e0202132 DOI 10.1371/journal.pone.0202132

13. Moschos MM, Gouliopoulos NS, Kalogeropoulos C, Androudi S, Kitsos G, Ladas D, Tsatsos M, Chatziralli I (2018) Psychological Aspects and Depression in Patients with Symptomatic Keratoconus. Journal of ophthalmology 2018: 7314308 DOI 10.1155/2018/7314308

14. Eliasi E, Bez M, Megreli J, Avramovich E, Fischer N, Barak A, Levine H (2021) The Association Between Keratoconus and Body Mass Index: A Population-Based Cross-Sectional Study Among Half a Million Adolescents. American journal of ophthalmology 224: 200-206 DOI 10.1016/j.ajo.2020.11.021

15. Naderan M, Rezagholizadeh F, Zolfaghari M, Naderan M, Rajabi MT, Kamaleddin MA (2015) Association between the prevalence of obstructive sleep apnoea and the severity of keratoconus. The British journal of ophthalmology 99: 1675-1679 DOI 10.1136/bjophthalmol-2015-306665

16. Gatzioufas Z, Thanos S (2008) Acute keratoconus induced by hypothyroxinemia during pregnancy. Journal of endocrinological investigation 31: 262-266 DOI 4517 [pii]

17. Lee R, Hafezi F, Randleman JB (2018) Bilateral Keratoconus Induced by Secondary Hypothyroidism After Radioactive Iodine Therapy. J Refract Surg 34: 351-353 DOI 10.3928/1081597x-20171031-02

18. Flasko Z, Zemova E, Eppig T, Modis L, Langenbucher A, Wagenpfeil S, Seitz B, Szentmary N (2019) Hypothyroidism is Not Associated with Keratoconus Disease: Analysis of 626 Subjects. Journal of ophthalmology 2019: 3268595 DOI 10.1155/2019/3268595

19. Spoerl E, Raiskup-Wolf F, Kuhlisch E, Pillunat LE (2008) Cigarette smoking is negatively associated with keratoconus. J Refract Surg 24: S737-740 DOI 10.3928/1081597x-20080901-18

20. Sahebjada S, Chan E, Xie J, Snibson GR, Daniell M, Baird PN (2021) Risk factors and association with severity of keratoconus: the Australian study of Keratoconus. Int Ophthalmol 41: 891-899 DOI 10.1007/s10792-020-01644-6

21. Seiler T, Huhle S, Spoerl E, Kunath H (2000) Manifest diabetes and keratoconus: a retrospective case-control study. Graefes Arch Clin Exp Ophthalmol 238: 822-825 DOI 10.1007/s004179900111

22. Hashemi H, Heydarian S, Hooshmand E, Saatchi M, Yekta A, Aghamirsalim M, Valadkhan M, Mortazavi M, Hashemi A, Khabazkhoob M (2020) The Prevalence and Risk Factors for Keratoconus: A Systematic Review and Meta-Analysis. Cornea 39: 263-270 DOI 10.1097/ico.0000000000002150

23. Kuo IC, Broman A, Pirouzmanesh A, Melia M (2006) Is there an association between diabetes and keratoconus? Ophthalmology 113: 184-190 DOI 10.1016/j.ophtha.2005.10.009

24. Bawazeer AM, Hodge WG, Lorimer B (2000) Atopy and keratoconus: a multivariate analysis. The British journal of ophthalmology 84: 834-836

25. Balasubramanian SA, Pye DC, Willcox MD (2013) Effects of eye rubbing on the levels of protease, protease activity and cytokines in tears: relevance in keratoconus. Clin Exp Optom 96: 214-218 DOI 10.1111/cxo.12038

26. Balasubramanian SA, Mohan S, Pye DC, Willcox MD (2012) Proteases, proteolysis and inflammatory molecules in the tears of people with keratoconus. Acta ophthalmologica 90: e303-309 DOI 10.1111/j.1755-3768.2011.02369.x
